# Tropical freshwater ecosystems have lower bacterial growth efficiency than temperate ones

**DOI:** 10.3389/fmicb.2013.00167

**Published:** 2013-06-21

**Authors:** André M. Amado, Frederico Meirelles-Pereira, Luciana O. Vidal, Hugo Sarmento, Albert L. Suhett, Vinicius F. Farjalla, James B. Cotner, Fabio Roland

**Affiliations:** ^1^Limnology Laboratory, Departamento de Oceanografia e Limnologia, Pós-graduação em Ecologia, Universidade Federal do Rio Grande do NorteNatal, Brazil; ^2^Departamento de Ecologia, Universidade Federal do Rio de JaneiroRio de Janeiro, Brazil; ^3^Departamento de Ecologia, Universidade Federal de Juiz de ForaJuiz de Fora, Brazil; ^4^Unidade de Biotecnologia e Ciências Biológicas, Centro Universitário Estadual da Zona OesteRio de Janeiro, RJ, Brazil; ^5^Ecology Evolution and Behavior Department, University of Minnesota, St. PaulMN, USA

**Keywords:** microbial metabolism, BGE, carbon cycle, tropical, temperate

## Abstract

Current models and observations indicate that bacterial respiration should increase and growth efficiency (BGE) should decrease with increasing temperatures. However, these models and observations are mostly derived from data collected in temperate regions, and the tropics are under-represented. The aim of this work was to compare bacterial metabolism, namely bacterial production (BP) and respiration (BR), bacterial growth efficiency (BGE) and bacterial carbon demand (BCD) between tropical and temperate ecosystems via a literature review and using unpublished data. We hypothesized that (1) tropical ecosystems have higher metabolism than temperate ones and, (2) that BGE is lower in tropical relative to temperate ecosystems. We collected a total of 498 coupled BP and BR observations (*N*_total_ = 498; *N*_temperate_ = 301; *N*_tropical_ = 197), calculated BGE (BP/(BP+BR)) and BCD (BP+BR) for each case and examined patterns using a model II regression analysis and compared each parameter between the two regions using non-parametric Mann–Whitney U test. We observed a significant positive linear regression between BR and BP for the whole dataset, and also for tropical and temperate data separately. We found that BP, BR and BCD were higher in the tropics, but BGE was lower compared to temperate regions. Also, BR rates per BP unit were at least two fold higher in the tropics than in temperate ecosystems. We argue that higher temperature, nutrient limitation, and light exposure all contribute to lower BGE in the tropics, mediated through effects on thermodynamics, substrate stoichiometry, nutrient availability and interactions with photochemically produced compounds. More efforts are needed in this study area in the tropics, but our work indicates that bottom-up (nutrient availability and resource stoichiometry) and top-down (grazer pressure) processes, coupled with thermodynamic constraints, might contribute to the lower BGE in the tropics relative to temperate regions.

## Introduction

Freshwater ecosystems are critical bioreactors in the global carbon cycle as they process a large fraction of the organic matter exported from terrestrial ecosystems (Cole et al., 2007; Tranvik et al., 2009). Heterotrophic bacteria play an important role in processing this organic matter and in releasing CO_2_ and inorganic nutrients (Cole et al., 1994; Odum et al., 2004), and re-integrating dissolved organic matter (DOM) to the food web through the microbial loop (Azam et al., 1983). Thus, bacterial production (BP), and bacterial respiration (BR) are key processes in the carbon cycle of all aquatic systems. Two parameters derived from BP and BR are useful tools to understand the role of bacteria in ecosystem functioning: bacterial carbon demand (BCD), which represents the total amount of carbon processed by bacteria (BCD = BP + BR) and bacterial growth efficiency (BGE), which is the proportion of carbon taken up by bacteria that is converted into biomass (BGE = BP/[BP + BR]) (for review see Del Giorgio and Cole, 1998). Higher BGE leads to higher energy and organic matter availability to higher trophic levels, i.e., the microbial loop. On the other hand, lower in BGE may result in higher carbon mineralization rates through CO_2_ production.

Several environmental factors may regulate bacterial metabolism and affect BGE and BCD. Water temperature is an important metabolic regulator and frequently correlates positively with BP, BR, and BCD, and negatively with BGE (Rivkin and Legendre, 2001; Biddanda and Cotner, 2002; López-Urrutia and Morán, 2007). Additionally, nutrient availability (such as N and P), DOM quality and stoichiometry, and bacterial predation pressure may also be important regulators of microbial activity (Farjalla et al., 2002, 2006; Hall and Cotner, 2007; Berggren et al., 2010; Vidal et al., 2011; Sarmento, 2012; Sarmento and Gasol, 2012). Considering that the tropical region (between latitudes 23°26′S and 23°26′N) presents higher sunlight incidence and mean temperature (Lewis, 1987, 1996) and that additional trophic levels within the microbial food web can persist throughout the year (Sarmento, 2012), one might expect that many aquatic processes, such as BGE and BCD could present differences between tropical and temperate regions.

The metabolic theory of ecology predicts that organisms living in warmer conditions exhibit higher metabolic rates than organisms living at lower temperatures (Brown et al., 2004). On the other hand, the ATP paradox suggests that higher metabolic rates yield lower biomass with higher energy dissipation (lower growth efficiency; Pfeiffer et al., 2001; Pfeiffer and Bonhoeffer, 2002; MacLean, 2008). Thus, it could be deduced that at the higher temperatures in the tropics BGE should be reduced relative to the temperate zones. This relationship of decreased BGE with increasing temperatures has been demonstrated already mostly within temperate ecosystems (Hall and Cotner, 2007; López-Urrutia and Morán, 2007; Berggren et al., 2010). Another recent study comparing tropical humic ecosystems to temperate models observed that BR was higher and more variable in the humic tropical systems, and also that BP tends to stabilize, relative to BR (Farjalla et al., 2009). These findings contradict other studies in temperate ecosystems that observed higher BP variability, rather than BR (Del Giorgio and Cole, 1998; Roland and Cole, 1999). Thus, following the humic-tropical pattern, BR would increase proportionally more in relation to BP, and the average BGE in the tropical ecosystems would be lower than in temperate systems, implying a lower relevance of the microbial loop in terms of biomass, but higher relevance in terms of carbon mineralization in tropical systems. However, a recent review on the role of the microbial food webs in tropical lakes was not able to demonstrate that BP was higher in the tropics, compared to temperate systems in small dataset (Sarmento, 2012).

Therefore, assuming higher and more constant average temperatures in tropical aquatic ecosystems compared to temperate aquatic ecosystems, some important questions arise regarding bacterial function in these ecosystem: (1) Do bacteria grow less efficiently in the tropics than in temperate ecosystems? (2) Is the humic-rich tropical ecosystem pattern relevant in a larger context of tropical ecosystems types? (3) If bacteria do grow less efficiently in the tropics than in temperate regions, is temperature the most important factor driving these differences?

In this paper we hypothesize that (1) tropical inland aquatic ecosystems have higher metabolic rates (e.g., BCD) than temperate ecosystems and, (2) that BGE is lower due to high respiration rates tropics. This paper aims to address and discuss these hypotheses by re-analyzing freshwater BP, BR and BGE data from literature (mainly from the temperate region), and by adding published and unpublished data from the tropical region. This work is an effort to expand current paradigms of microbial biogeochemistry to the tropical inland aquatic ecosystems. We point out the importance of increasing data production on BP and BR in the tropics to achieve a more accurate understanding about carbon cycling and the role of bacteria in this vast and understudied part of the world.

## Methods

We pooled models and bacterial production (BP) and bacterial respiration (BR) data from the literature (Table 1) and unpublished data from Lake Superior and northern Minnesota lakes (Sampled by J. Cotner and A. Amado), Swedish lakes (Sampled by L. Vidal and W. Granéli) and Amazonian lakes and rivers (Sampled by L. Vidal, G. Abril, F. Artigas and F. Roland). Lake Superior samples were taken at six sampling stations over five cruises in the western arm of the lake, from May–October 2006. Northern Minnesota lakes were sampled in July 2006 (summer) and January 2007 (winter) in a central point of each lake. Nineteen Swedish lakes were sampled in July 2007 (summer) in the deepest part of each lake over a DOC gradient from 3.7 to 26.8 mg L^−1^. Twenty Amazonian lakes and rivers were sampled in June and November (2009) at stations throughout the Amazon River basin (for details see Table 1). For the literature data we surveyed the papers used in the review by Del Giorgio and Cole (1998) and all papers available from 1998–2012 searching for “BGE” and “aquatic ecosystems” key-words in the ISI Web of Science (Table 1). We only used data from inland aquatic ecosystems where both BP and the correspondent BR rates were available. Literature data were extracted from tables and graphs and converted to microgram of carbon per liter per hour (μgC L^−1^ h^−1^) and log-transformed to perform statistical analysis. When the data were only available in graphs, they were extracted from the graphs using the Digitizeit and GraphClick softwares.

**Table 1:** **Database used in the analysis with the literature extracted and novel data**.

**References**	***N***	**Region**	**BR range (μgC L^−1^ h^−1^)**	**BP range (μgC L^−1^ h^−1^)**	**BGE range**	**BCD range (μgC L^−1^ h^−1^)**	**BR method**	**BP method**
Anesio et al. (2005)	5	Temperate	7.30–8.80	1.7–4.1	0.18–0.32	9.30–12.90	DIC increase	Leucine
Benner et al. (1995)	17	Tropical	3.12–22.68	0.31–2.70	0.02–0.34	4.59–25.38	Oxygen Winkler	Leucine/Thymidine
Berggren et al. (2007)	9	Temperate	2.78–4.09	0.54–2.36	0.12–0.44	3.84–6.15	DIC increase	Leucine
Berggren et al. (2009)	6	Temperate	2.88–7.96	0.63–2.63	0.18–0.26	3.50–10.58	DIC increase	Leucine
Berggren et al. (2010)	18	Temperate	0.32–4.05	0.27–1.48	0.24–0.56	0.58–5.33	DIC increase/Oxygen probe	Leucine
Biddanda and Cotner (2002)	14	Temperate	0.21–2.82	0.02–1.32	0.01–0.53	0.23–3.53	Oxygen Winkler	Leucine
Biddanda et al. (2001)	12	Temperate	0.23–3.66	0.02–0.82	0.06–0.39	0.28–4.04	Oxygen Winkler	Leucine
Cammack (2002)	28	Temperate	0.88–20.58	0.03–2.10	0.02–0.22	0.97–22.68	Oxygen Winkler	Leucine
Comte and Del Giorgio (2009)	6	Temperate	0.80–7.50	0.03–7.90	0.04–0.51	0.83–15.40	Oxygen MIMs	Leucine
Cotner and Amado Unpublished	30	Temperate	0.05–42.02	0.01–7.24	<0.01–0.50	0.11–49.25	Oxygen Microelectrodes	Leucine
Del Giorgio et al. (2006)	24	Temperate	2.53–11.56	1.00–3.96	0.14–0.38	3.89–14.95	Oxygen MIMs	Leucine
Farjalla et al. (2009)	24	Tropical	8.79–23.42	0.70–2.74	0.06–0.19	9.81–25.80	DIC increase	Leucine
Guillemette and Del Giorgio (2012)	11	Temperate	0.52–1.71	0.13–2.29	0.18–0.69	0.71–3.91	Oxygen MIMs	Leucine
Hall and Cotner (2007)	16	Temperate	0.37–12.82	0.11–2.14	0.04–0.62	0.48–13.31	Oxygen Winkler	Leucine
Kritzberg et al. (2005)	19	Temperate	0.68–16.91	0.05–1.01	0.01–0.34	0.85–17.08	Oxygen Winkler	Leucine
Lennon and Cottingham (2008)	5	Temperate	0.09–0.23	0.03–0.17	0.25–0.42	0.12–0.40	Oxygen Winkler	Leucine
Maranger et al. (2005)	34	Temperate	2.98–9.34	0.75–7.63	0.16–0.58	4.06–14.5	Oxygen Winkler	Leucine
Roland and Cole (1999)	19	Temperate	1.43–8.81	0.35–10.10	0.04–0.66	2.28–16.32	Oxygen Winkler	Leucine
Roland et al. (2011)	134	Tropical	0.92–67.98	0.07–9.71	0.01–0.40	0.99–71.34	Oxygen Winkler	Leucine
Schwaeter et al. (1988)	16	Temperate	6.31–38.94	3.12–8.46	0.21–0.45	10.15–38.94	Oxygen Winkler	Thymidine
Vidal and Granéli Unpublished	19	Temperate	5.01–83.23	0.02–0.48	<0.01–0.02	5.03–83.52	Oxygen Winkler	Leucine
Vidal et al. Unpublished	22	Tropical	1.56–51.53	0.03–3.45	<0.01–0.23	1.59–53.06	Oxygen Winkler	Leucine
Warkentin et al. (2011)	10	Temperate	6.57–190.00	0.13–14.40	0.01–0.26	6.70–195.40	Oxygen Probe	Leucine

BR, Bacterial respiration; BP, bacterial production; BGE, bacterial growth efficiency; BCD, bacterial carbon demand ranges display the minimum and maximum values from each study.

N_total_ = 498; N_tropical_ = 197; N_temperate_ = 301.

Regarding the original data presented in this work, BP rates were measured using the method of Smith and Azam, (1992; [^3^H]-leucine incorporation method). The incubation times varied between 0.5 and 5.0 h according to the local conditions (e.g., temperature, nutrients concentrations). BR rates were measured by dissolved oxygen consumption over a 24-h period. Oxygen concentrations were measured by different methods based on changes in dissolved oxygen concentrations over time. In samples from Lake Superior and from Minnesotan lakes we measured the changes in dissolved oxygen in the same sample over time (as initial and final) using a gold tip micro-probe connected to OXY-meter (Briand et al., 2004) controlled by the MicOx Software (Unisense©, Aarhus, Denmark). Samples from Amazonian ecosystems and Swedish lakes were measured in discrete samples at various time-points with sets of replicated flasks/vials using the Winkler technique and titrations with a potentiometric endpoint using a Mettler DL21 titrator (Granéli and Granéli, 1991). BR rates were transformed to carbon using a respiratory coefficient (RQ) of 1 (shown as being the most commonly used by Del Giorgio and Cole, 1998).

From the literature, we only considered BP data estimated by radioisotope incorporation (using tritium-labeled (^3^H) leucine or thymidine incorporation). As all BP estimates were measured over similar time scales, we considered that both methods estimated equivalent rates (Kirchman, 1992) and, thus, we did not apply any conversion factor between the results from the two methods. We did not consider BP data estimated from bacterial biomass accumulation in batch cultures. Despite the fact that it has been previously suggested that both radioisotope incorporation and biomass accumulation in batch cultures methods yield similar results (Del Giorgio and Cole, 1998), the latter method is employed at longer timescales and require grazers-free condition and the radioisotope incorporation methods represent conditions closer to those of the natural system.

We used literature BR data measured via three dissolved oxygen consumption methods [the two mentioned above plus by oxygen estimated by membrane inlet mass spectrometer (MIMS)] and through dissolved organic carbon (DIC) accumulation. The BR rates estimated as oxygen consumption were transformed to a carbon basis by respiratory coefficient (RQ) of 1, as in the description of the novel data above. BR rates are frequently estimated from filtered samples (over a very wide range of pore sizes, frequently from 0.45 to 3.0 μm), but also in unfiltered samples. In oligotrophic ecosystems BR accounts for up to 90% of plankton respiration (Biddanda et al., 2001) and, thus, it is acceptable that BR could even be measured in unfiltered water. For the purpose of this study, we used the corrections and assumptions of the authors in estimating BR from whole or filtered water measurements. As our aim was to compare tropical to temperate rates with the same criteria, we assumed that any discrepancies related to the filtration pore-size would be randomly distributed between the data from the two regions and would not affect the overall patterns.

The dataset presented here was constructed with data from inland aquatic ecosystems in both temperate and tropical regions (*N*_total_ = 498; *N*_tropical_ = 197; *N*_temperate_ = 301). Our dataset includes information mostly from lakes, rivers and reservoirs, excluding estuarine and marine data. To our knowledge, this is the largest dataset of BP and BR for freshwaters.

From BP and BR data, we calculated bacterial growth efficiency (BGE = BP/[BP + BR]) and bacterial carbon demand (BCD = BP + BR). Statistical analyses were performed in the R environment (www.r-project.org) using the “vegan” (Oksanen et al., 2008), “lmodel2” and “smart” packages. We performed model II linear regression using the major axis method (package “lmodel2” by Pierre Legendre) on log-transformed data between BP vs. BR, and BP vs. BGE from the whole data set and with tropical and temperate subsets. Slopes and intercepts for temperate and tropical subsets were compared using the “ma” function (package “smart” by David Warton) that tests hypotheses about slope or elevation (“elev.test”) based on confidence intervals comparison. We performed non-parametric Mann–Whitney Rank Sum test to compare median values of bacterial metabolism parameters between tropical and temperate subsets. Figures were made on SigmaPlot v.12 software (Systat Softare Inc.).

## Results

**Figure 1:**
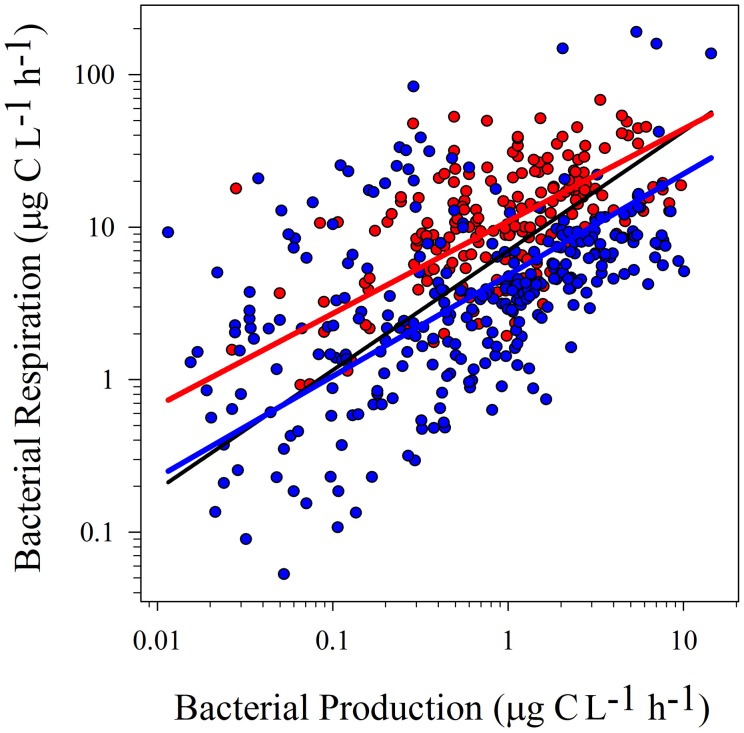
**Model II linear regressions between logBP and logBR from temperate (blue dots and blue line; *r*^2^ = 0.25; *p* < 0.0001), tropical (red dots and red line; *r*^2^ = 0.27; *p* < 0.0001) and the whole dataset (black line; *r*^2^ = 0.27; *p* < 0.0001).** See Table 2 for confidence intervals.

**Figure 2:**
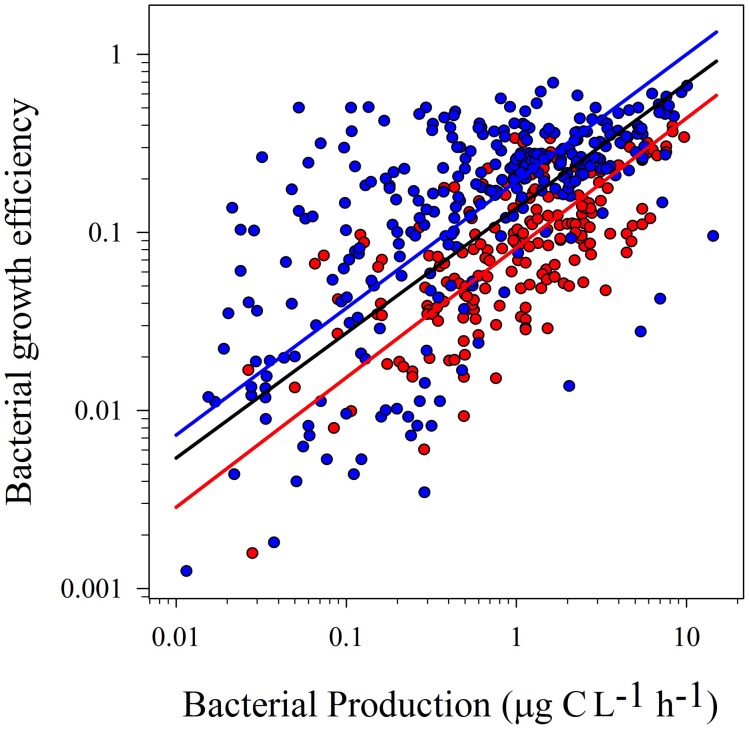
**Model II linear regressions between logBP and logBGE from temperate (blue dots and blue line; *r*^2^ = 0.41; *p* < 0.0001) and tropical (red dots and red line; *r*^2^ = 0.46; *p* < 0.0001) and the whole dataset (black line; *r*^2^ = 0.35; *p* < 0.0001).** See Table 2 for confidence intervals.

Model II linear regressions on log-transformed BP and BR were significant, either considering the whole dataset or data from tropical and temperate regions separately (Table 2; Figure 1). Similarly, there was a significant positive relationship between log-transformed BP and BGE (Table 2; Figure 2).

**Table 2:** **Slope, intercept and confidence interval (c.i.) of Type II Linear Regressions between log transformed BP vs. BR, and BP vs. BGE for tropical, temperate and whole dataset**.

	**Slope**	**95% [c.i.]**	**Intercept**	**95% [c.i.]**	***n***	***r*^2^**	***p***
**Log BR vs. Log BP**							
All data	0.78	[0.68: 0.90]	0.84	[0.83: 0.86]	498	0.27	<0.0001
Tropical	0.60	[0.47: 0.75]	1.04	[1.03: 1.04]	197	0.27	<0.0001
Temperate	0.66	[0.54: 0.80]	0.68	[0.66: 0.72]	301	0.25	<0.0001
**Log BGE vs. Log BP**							
All data	0.70	[0.62: 0.79]	−0.86	[−0.88: −0.85]	498	0.35	<0.0001
Tropical	0.73	[0.62: 0.85]	−1.09	[−1.09: −1.08]	197	0.46	<0.0001
Temperate	0.71	[0.62: 0.82]	−0.71	[−0.73: −0.69]	301	0.41	<0.0001

Statistical tests on confidence intervals (“ma” function from “smart” package) showed no significant differences between slopes for temperate and tropical sub-sets, for both regressions (logBR vs. logBP and logBGE vs. logBP). However, the confidence interval for the intercepts was lower in the tropical subset (Table 2). These results were confirmed testing the elevation confidence intervals (“ma” function from “smart” package), which showed significant differences between the intercepts from temperate and tropical subsets (logBR vs. logBP: *p* < 0.001, Test statistic: *t* = 14.73 with 195 degrees of freedom, logBGE vs. logBP: *p* < 0.001, Test statistic: *t* = −17.29 with 195 degrees of freedom), indicating that, at similar BP levels, BGE was lower and BR was, at least, two-fold higher in tropical systems.

**Figure 3:**
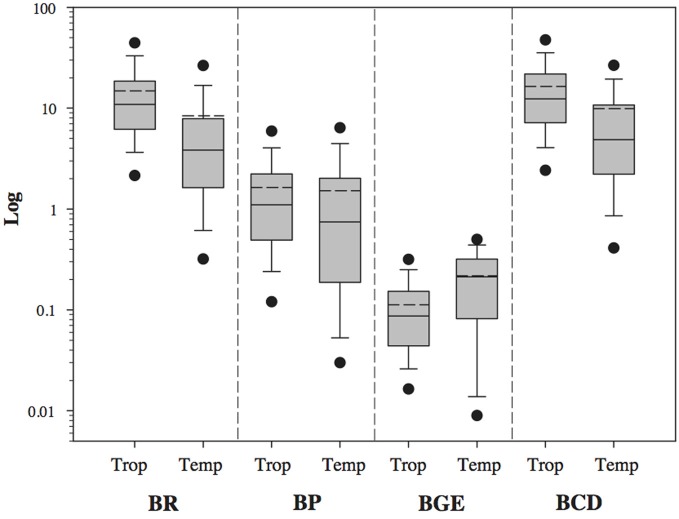
**Comparison of logBP, logBR, logBGE, and logBCD among temperate (Temp) and tropical (Trop) freshwater systems.** The central full line indicates the median value, the dotted line indicates the arithmetic mean value, the boxes indicate the lower and upper quartiles, the vertical lines indicate the 10th and 90th percentiles, and the dots represent the 5th and 95th percentiles. Tropical and temperate data were significantly different (non-parametric Mann–Whitney Rank Sum test) in all the four variables (BP, BR, BGE and BCD with *p* < 0.001, see text for details).

BP, BR and BCD presented higher median values in tropical than temperate subsets (logBR: *p* < 0.001, U Statistic = 13270.5; logBP: *p* < 0.001, U Statistic = 24013.5; LogBCD: *p* < 0.001, Mann–Whitney U Statistic = 15000.0; Figure 3), while the BGE median value was lower in the tropical subset (LogBGE: *p* < 0.001, U Statistic = 18352.0; Figure 3). It is worth noting that all parameters had a wider range of variation in temperate than the tropical subset (Figure 3).

## Discussion

This meta-analysis indicated that bacterial communities in tropical inland aquatic ecosystems had higher metabolic rates (BP, BR, and BCD) and lower BGE than temperate ecosystems. These results confirm the hypothesis presented here and the theoretical predictions from the literature (Lewis, 1987; Farjalla et al., 2009; Sarmento, 2012). We observed that BR rates were at least two fold higher in tropical ecosystems for a given BP, when compared to temperate ones. On one hand, heterotrophic bacteria process organic matter at faster rates in tropical ecosystems, regenerating inorganic nutrients more rapidly, but they do so while converting a smaller proportion of the organic matter into biomass. Even though we observed higher BP in the tropics, BR increased relatively more, resulting in lower BGE.

Higher BR rates in the tropics (Figures 1, 3) indicate that, proportionally, more energy is required to maintain a similar bacterial biomass, when compared to temperate ecosystems. In other words, similar bacterial biomass could recycle a greater quantity of carbon in tropical ecosystems compared to temperate ones. Additionally, previous studies reported a comparatively lower bacterial abundance in tropical systems along a productivity gradient relative to temperate systems (Roland et al., 2010; Sarmento, 2012), reinforcing the idea of lower BGE in the tropics. Thus, regarding the allegedly important role of the microbial food web in energy and nutrient regeneration, (for review see Del Giorgio and Cole, 1998), we argue that remineralization is greatly emphasized in tropical inland aquatic ecosystems.

For the first time, we present here evidence of consistent differences in bacterial metabolic rates between tropical and temperate freshwater ecosystems, with a large and representative dataset. These observations were hypothesized in past tropical limnology literature (Lewis, 1987; Farjalla et al., 2009; Sarmento, 2012), but never demonstrated at large spatial scales. Lower BGE in the tropics is likely due in part to two factors that differ greatly between the tropics and the temperate regions: (1) higher average solar irradiance in the tropics and (2) fundamental differences in trophic structure. It is well established that the higher sunlight irradiance in aquatic ecosystems directly affects water temperature, substrate stoichiometry (e.g., higher C:N, C:P under higher light incidence; see Sterner et al., 1998) and nutrient availability (such as N and P). In turn, these factors are important regulators of bacterial activity in aquatic systems (Del Giorgio and Cole, 1998) and can have effects on metabolic efficiency (i.e., BGE) under different environmental conditions (Hall et al., 2009).

Temperature is considered a critical environmental factor affecting microbial metabolism and seems to play a major role in different aspects, from physiology to community structure and algal excretion rates. Recent studies have shown that temperature positively correlates with BP and BR, but negatively with BGE, especially in systems where nutrients (N and P) are not limiting (Rivkin and Legendre, 2001; López-Urrutia and Morán, 2007; Berggren et al., 2010). Usually, under nutrient limitation, BGE is already low (Berggren et al., 2010). Furthermore, it has been shown that the C:P ratio of heterotrophic bacterial biomass increases with increasing temperature even with no change in growth rates (Cotner et al., 2006). Moreover, bacteria with high biomass C:P ratio, as occur under strong P-limitation conditions present relatively lower BGE than when in low biomass C:P ratio (Phillips, 2012). Therefore, it seems reasonable to conclude that the lower BGE in the tropics could result from higher average temperatures coupled with stronger nutrient, i.e., P limitation.

Previous studies predicted that nutrient recycling rates might be twice as high in tropical than in temperate ecosystems (Lewis, 1987). Our results corroborate this prediction showing that BR rates per BP unit was at least two fold higher in the tropics, consistent with higher temperatures and/or more nutrient-limited growth in the tropics. Although nutrient-limited growth has also been demonstrated in many temperate ecosystems as well, it is possible that the consistently higher BGE observed there could be due in part to less extreme nutrient limitation and perhaps stronger limitations by organic carbon availability (e.g., lower C:P ratios in primary producers). So, although nutrient regeneration rates may be high in warmer conditions, it is also likely that increasing growth demands and competition with autotrophs makes it more likely that bacteria in the tropics are limited by inorganic nutrients (Downing et al., 1999; Flecker et al., 2002).

Physical constraints of a thermodynamic nature should also be considered in order to explain the patterns observed. Considering that ATP production supports most biosynthetic assemblies (De Duve, 1991), a trade-off between rate and yield of ATP production might have important consequences to metabolism (Pfeiffer et al., 2001; Pfeiffer and Bonhoeffer, 2002; Schuster et al., 2008). The ATP paradox suggests that nature does not select for increased molar yield (detailed discussion in Schuster et al., 2008), because the highest thermodynamic efficiencies do not correlate to the highest growth rates (Westenhoff et al., 1983; Pfeiffer et al., 2001; Pfeiffer and Bonhoeffer, 2002) and this was confirmed in natural bacterial communities in aquatic ecosystems (Del Giorgio and Cole, 1998; Carlson et al., 2007). Thus, low metabolic rates lead to more efficient growth, while high metabolic rates (faster growth) results in low efficiency of biomass production (MacLean, 2008). For ecosystems, one of the implications is that one would expect cooler, more structured ecosystems where environmental changes are more predictable to have higher trophic efficiencies (Pfeiffer et al., 2001; Pfeiffer and Bonhoeffer, 2002); and lower efficiencies are expected in warmer, high metabolism ecosystems. Our observations are consistent with these predictions.

The tropics may also contrast with the temperate zone in terms of how sunlight interacts with the terrestrially-derived DOM. Assuming similar DOM export from terrestrial ecosystems and similar DOM concentrations in aquatic ecosystems at both tropical and temperate regions, greater photo-exposure in the tropics should increase production of reactive oxygen species (ROS) (Zepp and Cline, 1977; Cory et al., 2009). These compounds, such as singlet oxygen and hydrogen peroxide typically reduce microbial metabolism (Cory et al., 2010) by affecting cell structures activating repair mechanisms increasing the energy expenses (Madigan et al., 2010), and reducing BGE. ROS may also consume biologically available substrates, such as some amino acids, reducing bacterial growth and potentially BGE (Amado et al., 2007; Cory et al., 2010). Thus, the effects of higher DOM photochemical degradation and ROS production on microbial metabolism might be more pronounced in the tropics, also contributing to lower BGE (Figures 1, 3) due to higher sunlight irradiance throughout the year.

In studying global phenomena, such as using meta-analysis, one needs to be cautious to avoid misinterpretation of data as a consequence of methodological artifacts. Regarding BR data, one could argue that using RQ equal to 1 to convert BR from oxygen consumption to carbon basis is not adequate to a large dataset with distinct ecosystems from different regions, because it has recently been shown that RQ can vary between 0.8 and 1.4 depending on the origin and oxidation state of the DOM (Berggren et al., 2011). However, without further information about each ecosystem and/or differences in RQs between the temperate and tropical regions, applying a global RQ of 1 seems to be the most logical and conservative solution. Nonetheless, the highest RQ values have been observed when bacteria use DOM compounds that are highly oxidized, such as photochemically degraded DOM (Berggren et al., 2011). Taking into account that tropical ecosystems are exposed to higher sunlight incidence (Lewis, 1987, 1996) and that DOM is more photochemically oxidized compared to temperate ecosystems (Farjalla et al., 2009), we should expect the highest RQ in the tropical bacterial communities. Higher RQ values would increase BR rates in tropical ecosystems even more, and lower BGE.

Finally, biotic interactions might also contribute to explain the differences observed in bacterial metabolism along the latitudinal gradient. The fact that tropical ecosystems have slightly higher BP and lower bacterial abundance (Roland et al., 2010; Sarmento, 2012) is a strong indication that bacterial loss factors (such as grazing by protists or virus-induced lysogeny) might be more relevant at low latitudes. Despite the few data available for tropical lakes, recent studies provided strong indications that grazing on bacteria by microzooplankton (mainly heterotrophic nanoflagellates) in tropical lakes should be relatively high (Tarbe et al., 2011; Sarmento, 2012). This expected high grazing pressure on bacteria would explain the lower bacterial abundances in the tropics along the productivity gradient (Roland et al., 2010; Sarmento, 2012). Alternatively, strong grazing control might likely maintain bacterial communities in a rapid growth condition (e.g., as in the exponential growth phase), resulting in high-energy metabolic expenditure (such as in the ATP paradox, see discussion above). Concerning viral infection, there is almost no information available on how viral infection varies with latitude or temperature, but it has been suggested that higher bacterial metabolic rates are usually associated with low viral induced lysogeny (Maurice et al., 2010).

## Concluding remarks

Including data from tropical freshwaters in a conventional (mainly temperate) bacterial metabolism database did not change radically the relationships among BP, BR and BGE previously reported. However, a comparative analysis of tropical against temperate data indicated that BP, BR, and BCD were higher in tropical than in temperate ecosystems. Moreover, comparing both regions, the difference was more pronounced in BR rates than in BP, and consequently BGE in tropical ecosystems was lower. Furthermore, BR rates per BP unit were at least two fold higher in the tropics than in temperate ecosystems indicating higher nutrients turnover rates. Higher annual temperatures, increased nutrient limitation and different food web configurations all help to explain the higher energy dissipation in tropical regions. Lastly, higher sunlight exposure in the tropics contributes to higher photochemical DOM degradation and oxidation states and higher photochemical production of ROS. The current model of bacterial metabolism based on the relationship of BR and BP (Del Giorgio and Cole, 1998) is not adequate to predict the relationships between these two metabolic parameters efficiently at low latitudes. The addition of a large set of tropical data did not improve the general model, but highlighted some relevant factors that regulate microbial metabolism. Finally, we point out the need for intensive data collection on this topic, particularly in tropical regions in multiple biomes and ecosystem types.

### Conflict of interest statement

The authors declare that the research was conducted in the absence of any commercial or financial relationships that could be construed as a potential conflict of interest.
